# miR-200b Precursor Can Ameliorate Renal Tubulointerstitial Fibrosis

**DOI:** 10.1371/journal.pone.0013614

**Published:** 2010-10-25

**Authors:** Shigeyoshi Oba, Shintaro Kumano, Etsu Suzuki, Hiroaki Nishimatsu, Masao Takahashi, Hajime Takamori, Masatoshi Kasuya, Yousuke Ogawa, Kenichiro Sato, Kenjiro Kimura, Yukio Homma, Yasunobu Hirata, Toshiro Fujita

**Affiliations:** 1 Department of Nephrology and Endocrinology, University of Tokyo School of Medicine, Tokyo, Japan; 2 Department of Urology, University of Tokyo School of Medicine, Tokyo, Japan; 3 Institute of Medical Science, St. Marianna University School of Medicine, Kawasaki, Japan; 4 Department of Cardiovascular Disease, University of Tokyo School of Medicine, Tokyo, Japan; 5 Department of Nephrology and Hypertension, St. Marianna University School of Medicine, Kawasaki, Japan; University of Pittsburgh Medical Center, United States of America

## Abstract

Members of the miR-200 family of micro RNAs (miRNAs) have been shown to inhibit epithelial-mesenchymal transition (EMT). EMT of tubular epithelial cells is the mechanism by which renal fibroblasts are generated. Here we show that miR-200 family members inhibit transforming growth factor-beta (TGF-beta)-induced EMT of tubular cells. Unilateral ureter obstruction (UUO) is a common model of EMT of tubular cells and subsequent tubulointerstitial fibrosis. In order to examine the role of miR-200 family members in tubulointerstitial fibrosis, their expression was investigated in the kidneys of UUO mice. The expression of miR-200 family miRNAs was increased in a time-dependent manner, with induction of miR-200b most pronounced. To clarify the effect of miR-200b on tubulointerstitial fibrosis, we injected miR-200b precursor intravenously. A single injection of 0.5 nM miR-200b precursor was sufficient to inhibit the increase of collagen types I, III and fibronectin in obstructed kidneys, and amelioration of fibrosis was confirmed by observation of the kidneys with Azan staining. miR-200 family members have been previously shown to inhibit EMT by reducing the expression of *ZEB-1* and *ZEB-2* which are known repressors of E-cadherin. We demonstrated that expression of *ZEB-1* and *ZEB-2* was increased after ureter obstruction and that administration of the miR-200b precursor reversed this effect. In summary, these results indicate that miR-200 family is up-regulated after ureter obstruction, miR-200b being strongly induced, and that miR-200b ameliorates tubulointerstitial fibrosis in obstructed kidneys. We suggest that members of the miR-200 family, and miR-200b specifically, might constitute novel therapeutic targets in kidney disease.

## Introduction

Micro RNAs are small non-coding RNA molecules that can regulate gene expression by interacting with multiple mRNAs and inducing either translational suppression or degradation of mRNA. Recently, several miRNAs have been implicated in regulating the initial step in epithelial-mesenchymal transition (EMT). Several reports have shown that members of the miR-200 family (miR-200a,b,c, miR-141 and miR-429) inhibit EMT through direct targeting of *ZEB1* and *ZEB2*, which encode transcriptional repressors of E-cadherin in kidney tubular cells [1], breast cancer cells [2], and mammary epithelial cells [3]. Recent reports have indicated that a double-negative feedback loop between *ZEB1*, *ZEB2* and miRNA-200 family members regulates EMT in kidney tubular cells [4].

In addition to being an early step in tumor metastasis, EMT is known to be associated with many pathophysiological situations, such as formation of various tissues during embryonic development [5], and of keratinocytes during wound healing [6]. In the kidneys, EMT of tubular epithelial cells is a mechanism by which renal fibroblasts are generated, and the importance of EMT has been demonstrated in experimental models, where blockade of EMT attenuates renal fibrosis. Renal fibrosis correlates with decline of renal function and is one of the causes of impaired renal function. The inhibition of EMT of tubular epithelial cells therefore represents a possible novel therapeutic approach to counteract the progression of renal disease [7], [8]. A common model of renal tubulointerstitial fibrosis is the mouse model of unilateral ureter obstruction (UUO) [9]. Given their ability to inhibit EMT, we investigated whether injection of miR-200 miRNA family precursors - chemically modified double strand of RNA which form RNA-induced silencing complex (RISC) like complex and can be processed by endonuclease Dicer into mature miR-200 family in cells - could ameliorate tubulointerstitial fibrosis by inhibition of EMT of tubular epithelial cells in UUO model mice.

## Materials and Methods

### Western blotting of E-cadherin and N-cadherin

Western blotting analysis of E-cadherin and N-cadherin was performed in HK-2 cells stimulated with 10 ng/ml transforming growth factor-beta (TGF-beta) for 24, 48 and 72 hours. To investigate the effect of micro RNAs on EMT, HK-2 cells were transfected with 20 pmol/ml miR-200 family precursors for 24 hours using Lipofectamine RNAiMax (Invitrogen), then stimulated with 10 ng/ml TGF-beta. After 24 hours the expression of E-cadherin and N-cadherin was investigated with western blotting. Western blotting analysis was performed following. Ten micro gram of protein extracts were separated on 10% SDS-polyacrylamide gels and transferred onto nylon membranes (Millipore Corp., Bedford, MA) using a semidry blotting system (Amersham Pharmacia Biotech, Uppsala, Sweden). After blocking in 1× PBS, 5% nonfat dry milk, 0.2% Tween 20 at 4 °C overnight, the membranes were incubated with the primary antibodies in blocking buffer (1× PBS, 2% nonfat dry milk, 0.2% Tween 20) for 1 h at room temperature. Antibodies were used at a dilution of 1∶300. The membranes were washed three times with the blocking buffer and then incubated with secondary antibodies, which were conjugated with horseradish peroxidase (Amersham Pharmacia Biotech, Buckinghamshire, United Kingdom) at a final dilution of 1∶7,000. After final washes with 1× PBS, 0.2% Tween 20, the signals were detected using ECL chemiluminescence reagents (Amersham Pharmacia Biotech). Antibodies; E-cadherin, mouse monoclonal antibody anti-E-cadherin (BD Bioscience), N-cadherin, mouse monoclonal antibody anti-N-cadherin (BD Bioscience). To confirm that the same amount of protein was investigated, the expression of beta-actin was investigated simultaneously. All experiments were performed in triplicate.

### Micro RNA assays

Total RNA was extracted using the *miR*Vana miRNA isolation kit (Ambion). For micro RNA assays, mature miRNAs were reverse transcribed using a specific adapter, and real-time PCR was performed *Taq*Man micro RNA assays (Applied Biosystems). All data were normalized to U6 expression.

### Injection of miR-200b precursor

All procedure used in this study were approved by the Ethics Committee for the Use of Experimental Animals in Tokyo University. All possible efforts were made to minimize animal suffering and the numbers of animal used. The renal press-mediated transfection method was used to efficiently deliver the miR-200b precursor to the kidney [10]. 7 week old Balbc mice were intravenously injected with 0.5 nM miR-200b precursor (Ambion), immediately followed by pressing the left kidney. As a control experiment, 0.5 nM negative control micro RNA precursor (Ambion) and Cy3 dye-labeled Pre-miR control (Ambion) were intravenously administered as above.

### Semiquantitative Evalutation of fibrosis

To investigate fibrosis in the UUO model mice, the kidneys were harvested and fixed with 4% paraformaldehyde. 3microm paraffin section was stained with Azan stating. The slides were coded and examined in a blind fashion. Each kidney specimen were graded according to the extent of fibrosis by using the method previously described [11]. Grades were assigned to each slide according to the following criteria. Grade of fibrosis: 0, no fibrosis; 1, mild fibrosis (10 to 30% in area); 2, moderate fibrosis (30% to 50% in area); 3, severe fibrosis (>50% in area).

### Real-time PCR of type I, III collagen, fibronectin, E-cadherin, ZEB-1 and ZEB-2

Real-time PCR was performed to measure the expression of type I,III collagen, fibronectin, ZEB-1, ZEB-2 and E-cadherin of UUO day-6 kidneys. The data are normalized to beta-actin mRNA and shown as mean±S.E.(n = 8).Real-time PCR was performed with following PCR primers: type I collagen, 5′-TCCTGGCAACAAAGGAGACA-3′ and 5′-GGGCTCCTGGTTTTCCTTCT-3′; type III collagen, 5′-ACGTAGATGAAxTTGGGATGCAG-3′ and 5′-GGGTTGGGGCAGTCTAGTC-3′; Fibronectin, 5′-AGACCATACCTGCCGAATGTAG-3′ and 5′-GAGAGCTTCCTGTCCTGTAGAG-3′; E-cadheirn, 5′-GCACTCTTCTCCTGGTCCTG-3′ and 5′-TATGAGGCTGTGGGTTCCTC; ZEB1, 5′-TGGCAAGACAACGTGAAAGA-3′ and 5′-AACTGGGAAAATGCATCTGG-3′; ZEB2, 5′-TAGCCGGTCCAGAAGAAATG-3 and 5′-GGCCATCTCTTTCCTCCAGT-3′.

### Immunofluorescence studies

To detect the change of the expression of vimentin and cytokeratin 18 in the kidneys of UUO by miR-200b precursor, we examined the localization of them with immunofluorescein staining. The paraffin sections of kidneys were incubated with rabbit monoclonal antibody anti-vimentin (EPOT MICS) or mouse monoclonal anti-cytokeratin 18 (PROGEN) for 1 hr. The sections were washed with PBS and incubated with the FITC-conjugated anti-rabbit IgG antibody (DAKO cytomation) (1∶50) or the Hilyte Fluor 555-labeled anti-mouse IgG antibody (Ana Spec, Inc.) (1∶50) for 30 min, washed with PBS and observed under the Confocal Scanner Unit.

### ZEB1 and ZEB2 3′-UTR Luciferase Reporter Assays

The 3′-UTR**s** for both *ZEB1* and *ZEB2* were PCR-amplified from genomic DNA. PCR primers used to amplify the Zeb1 3′-UTR include 5′-AAAAATCCGGGTGTGCCTGA-3′ (forward) and 5′-AACTGCTTTCTACTGCTCTG-3′ (reverse), whereas the primers used to amplify the Zeb-2 3′-UTR include 5′-CAGTTCAGCCAAGACAGAGT-3′ (forward) and 5′-TTCGAGCATGGTCATTTTC-3′UTR (reverse). Amplified 3′-UTRs were cloned downstream of the luciferase coding region in the pGL-3 control (Clontech). HK-2 cells were seeded in 6-well plates 24 hr prior to transfection. 2.5microg of reporter plasmid along with 2.5 microg of control *Renilla*-luciferase plasmid were co-transfected using Lipofectamine LTX(Invitrogen). To assess the effect of miR-200 family precursor on reporter activity, 50 pM of synthetic precursor miRNAs (pre-miRs) (Ambion) were co-transfected. All experiments were performed in triplicate.

### Statistical Analysis

All data are reported as mean ± S.E. When comparisons were made between two different groups, statistical significance was determined by the Student's t-test using the Stat View software program.

## Results

**Figure 1:**
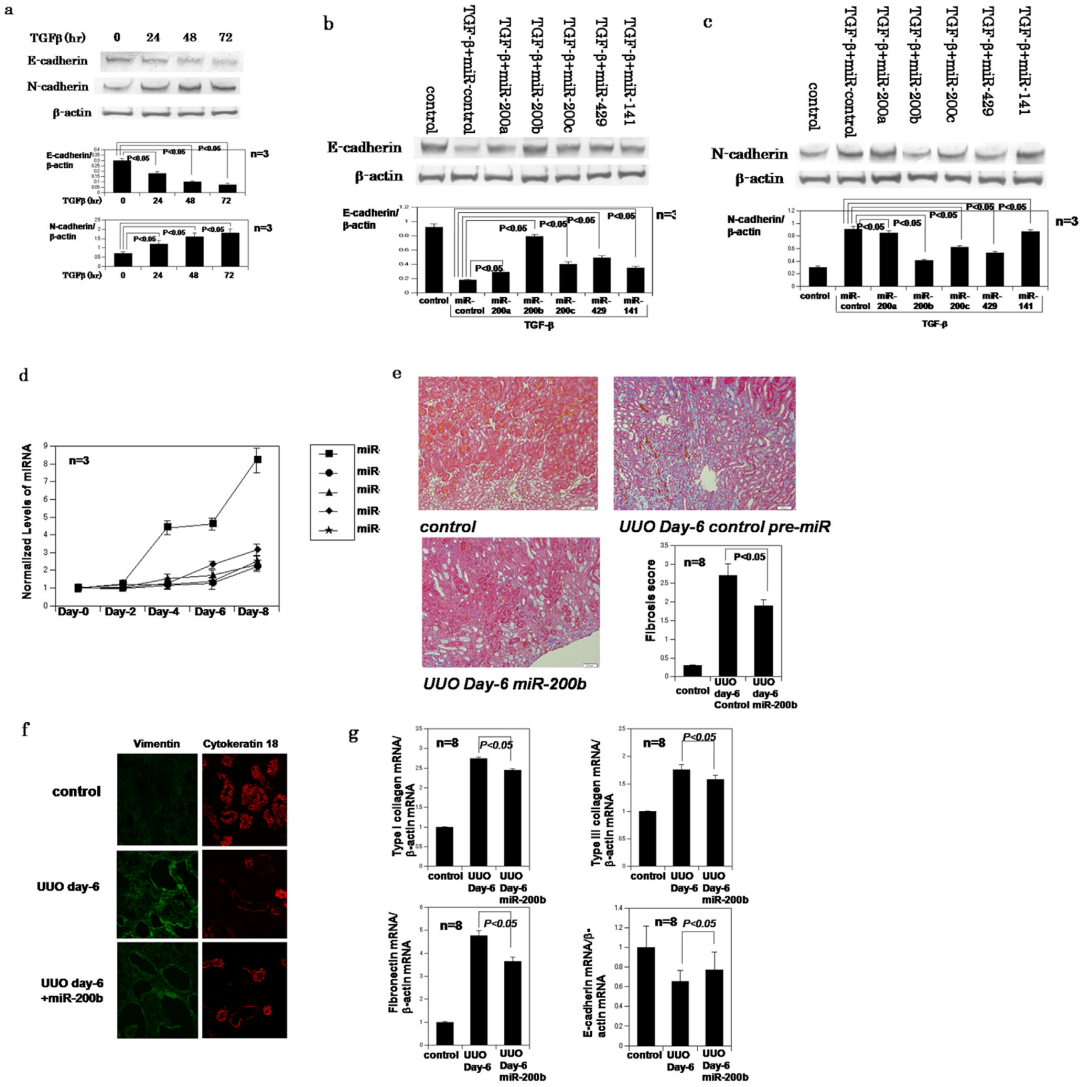
miR-200 family members can ameliorate EMT and tubulointerstitial fibrosis in UUO model mouse kidneys. **a**, Western blotting analysis confirms down-regulation of E-cadherin and up-regulation of N-cadherin during EMT. **b**,**c**, Western blotting of E-cadherin and N-cadherin in HK-2 cells treated with TGF-beta and transfected with control miR or miR-200 members precursors individually. In western blotting, each band has been quantitated and subjected to densitometry to determine if statistically significant difference exists between groups **d**, Changes in miR-200 family levels in UUO model mice kidneys, as measured by *TaqMan* qRT-PCR and normalized to U6 expression. The data are means from a representative time course experiment measured in triplicate and are presented as mean±S.E. **e**, Azan staining of intact Balbc mouse kidney, UUO day-6 kidneys after transfection of negative control pre-miR or miR-200b precursor. Fibrosis score; The tubulointerstitial fibrosis score in kidneys of miR-200b precursor injected group was significantly lower than of control group: *P*<0.05, The data are means from experiment measured in triplicate and are presented as mean±S.E (n = 8) **f**, Immunofluorescence studies of vimentin and cytokeratin 18 in the kidneys of UUO day6 mice and UUO day6 mice injected by miR-200b precursor. miR-200b ameliorates up-regulation of vimentin and down-regulation of cytokeratin 18. **g**, real-time PCR confirms that miR-200b precursor reverses up-regulation of type I,III collagen and fibronectin of UUO day-6 kidneys. The data are normalized to beta-actin mRNA and shown as mean±S.E.(n = 8).

Initial western blotting analysis demonstrated TGF-beta-mediated repression of E-cadherin, and induction of N-cadherin, in HK-2 kidney tubular cells (Fig. 1a). All miR-200 family precursors tested were capable of inhibiting the reduction of E-cadherin and up-regulation of N-cadherin affected by TGF-beta in HK-2 cells (Fig. 1b, c).

We next investigated the role of miR-200 family members in EMT of tubular cells in UUO mice. Micro RNA assays showed that expression of all members of the miR-200 family tested were increased in a time-dependent manner after ureter obstruction (Fig. 1d); the induction of miR-200b was most pronounced. Since the most efficient *in vitro* inhibition of TGF-beta was mediated by the miR200-b miRNA (Fig. 1b and Fig. 1c) we next evaluated its effect *in vivo* by injecting miR-200b precursor via the abdominal vein prior to occlusion of the left ureter. A single injection of 0.5 nM miR-200b precursor was sufficient to ameliorate tubulointerstitial fibrosis in the kidney 6 days after UUO (Fig. 1e), an observation confirmed by real time PCR analysis of type I collagen, type III collagen and fibronectin and Azan staining (Fig. 1e). To demonstrate that reduction of ZEB1 and ZEB2 levels reduces epithelial to mesenchymal transition, tissue stains for epithelial makers, cytokeratin 18 and for mesenchymal markers, vimentin in animals treated with miR-200b and control animals have shown in Figure 1f.

**Figure 2:**
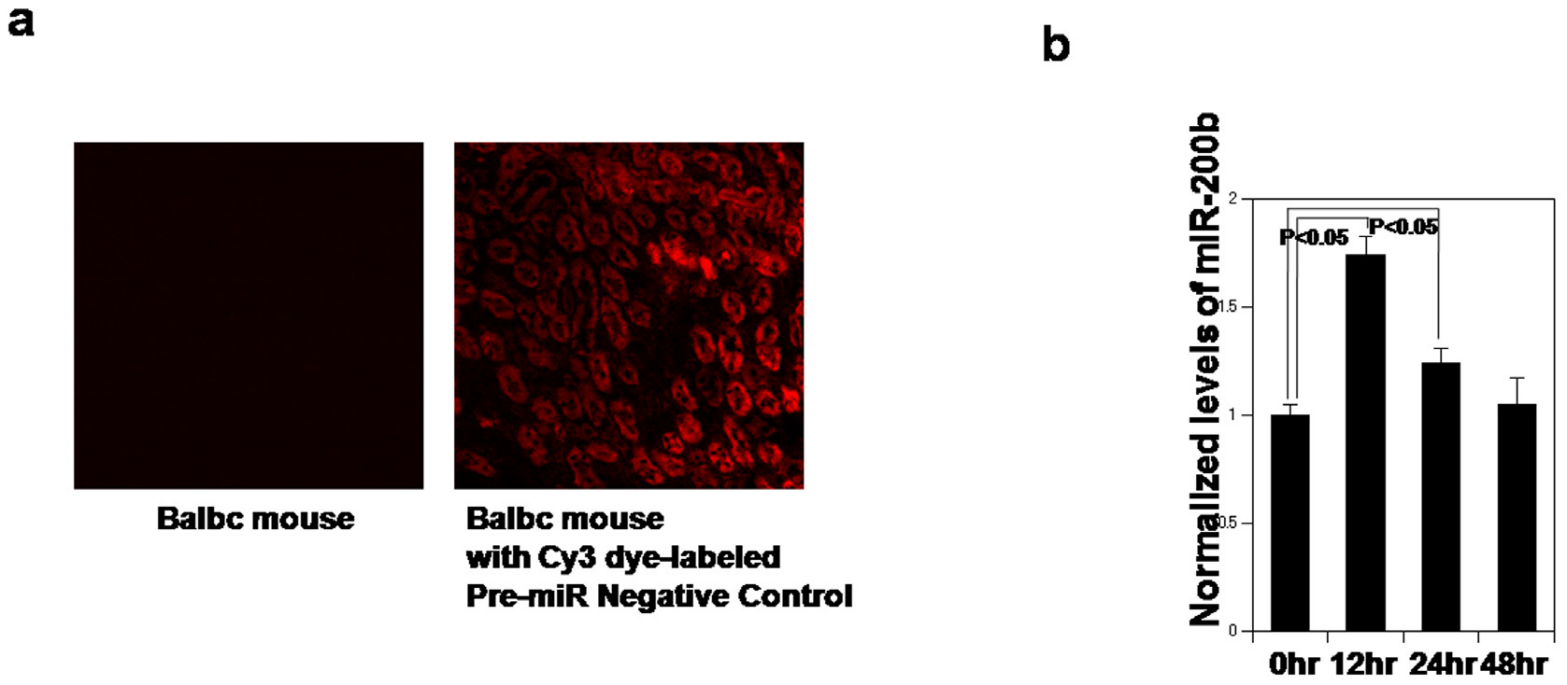
Injected miR-200b precursor can be converted to mature miR-200b in kidneys. **a**. Laser microscopy images of kidney, frozen sections at 24 hr after intravenously administration of Cy3 dye-labeled Pre-miR control. **b**. Changes in miR-200 family levels in Balbc mice kidneys 12, 24, 48 hours after intravenously administration of miR-200b precursor, as measured by *TaqMan* qRT-PCR and normalized to U6 expression. The data are means from experiment measured in triplicate and are presented as mean±S.E. *P*<0.05: kidneys 12, 24 hours after intravenously administration of miR-200b precursor compared with 0 hour.

Laser microscopic observation revealed that injected Cy3 dye-labeled Pre-miR control could be observed in tubular cells (Fig. 2a). Micro RNA assays revealed that injected miR-200b precursor can be converted to mature miR-200b in mouse kidneys (Fig. 2b)

**Figure 3:**
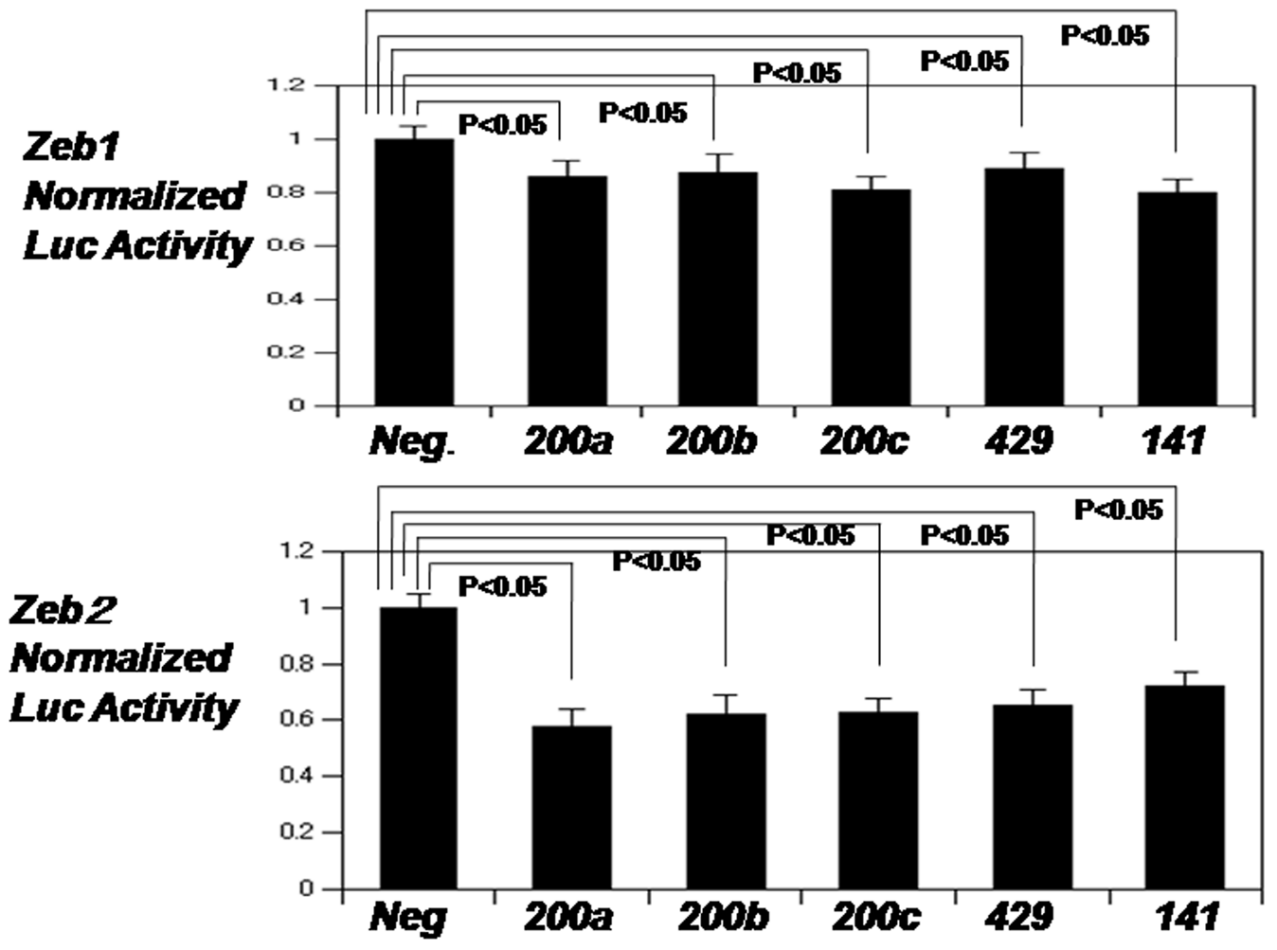
miR-200 family members target the transcriptional repressors ZEB1 and ZEB2. Normalized activity of luciferase reporter with the ZEB1 or ZEB2 3′UTR in HK-2 cells in the presence of co-transfected negative control pre-miR or miR-200 family individually. Luciferase activity was measured after 24 hours. The data are mean±S.E. of triplicates and are shown as the ratio of firefly to Renilla luciferase activity. *P*<0.05: HK-2 cells in the presence of co-transfected miR-200 family compared with negative control pre-miR

Previous studies showed that inhibition of EMT by miR-200 family members was mediated by their inhibition of the expression of the E-cadherin repressors *ZEB1* and *ZEB2* through binding to the 3′-UTR region of ZEB1 and ZEB2 mRNAs [1]–[3]. In order to confirm that miR-200 family members target ZEB1 and ZEB2 3′-UTRs, we co-transfected either ZEB1-3′UTR-luciferase or ZEB2-3′UTR-luciferase reporter vectors with miR-200 family precursors in HK-2 cells. The luciferase activity of both reporters was significantly reduced in the presence of all miR-200 family members tested (Fig. 3).

**Figure 4:**
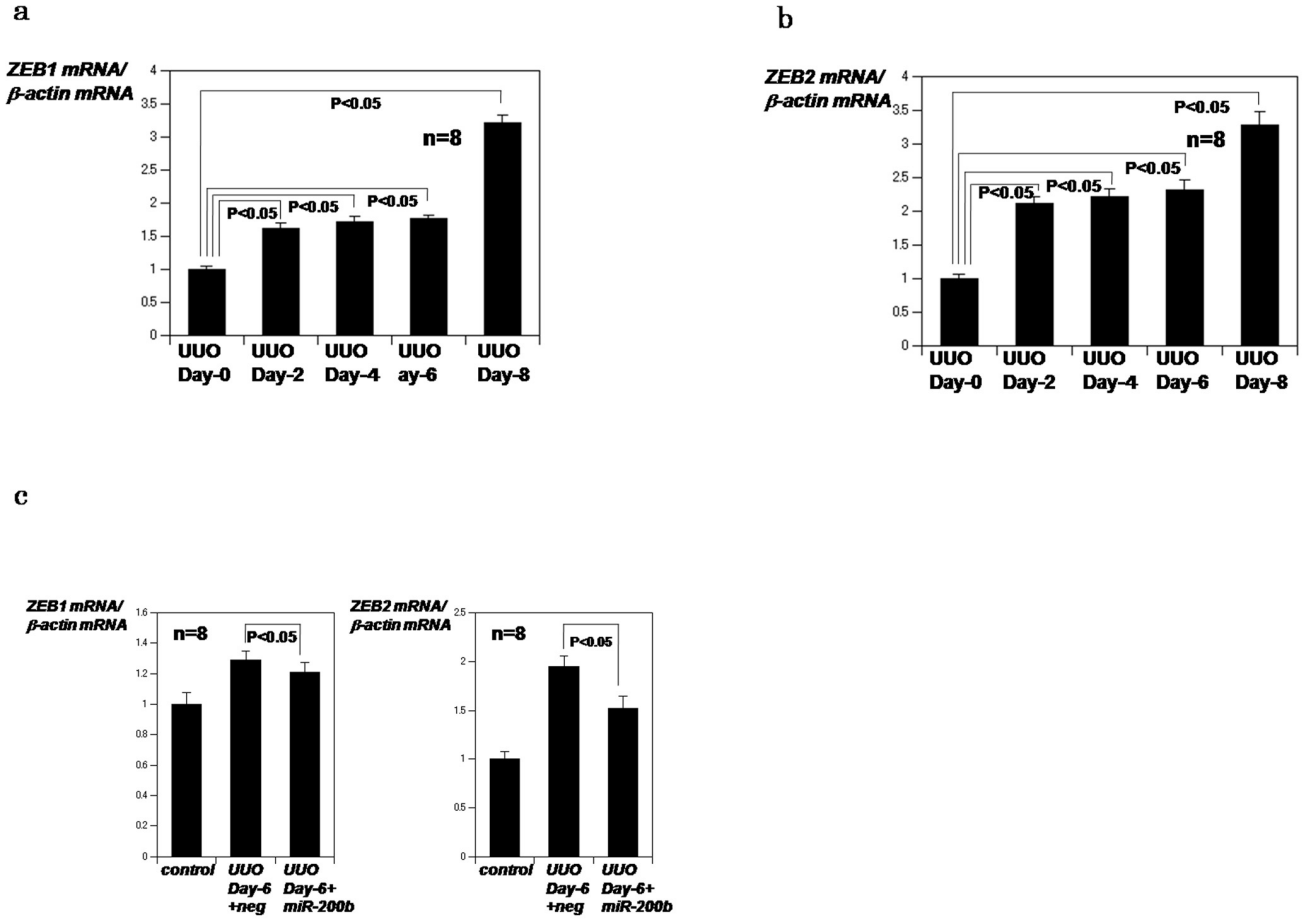
ZEB1 and ZEB2 expression in UUO model mice. **a**,**b**, Real time PCR analysis of ZEB1 or ZEB2 mRNA in kidneys **obstructed for 2,4,6 or 8** days. The data are normalized to beta-actin mRNA and shown as mean±S.E.(n = 8). *P*<0.05: kidneys obstructed for 2,4,6 and 8 compared with 0 day **c**, Real time PCR analysis of ZEB1 or ZEB2 mRNA in **obstructed for 6 days** after i.v. administration of miR-200b precursor. The data are normalized to beta-actin mRNA and shown as mean±S.E.(n = 8). *P*<0.05: **obstructed for 6 days** after i.v. administration of miR-200b precursor compared with negative control pre-miR.

We next investigated whether expression of ZEB1 and ZEB2 increased as a result of kidney obstruction. Real time PCR analysis confirmed that ZEB1 and ZEB2 were induced in a time-dependent manner after obstruction (Fig. 4a, b), an effect reversed by injection of miR-200b precursor (Fig. 4c). These results indicate that members of the miR-200 family of miRNAs are induced after ureter obstruction and that miR-200b ameliorates tubulointerstitial fibrosis in obstructed kidneys.

## Discussion

In this report, we show that members of the miR-200 family of miRNAs are induced after ureter obstruction and that miR-200b ameliorates tubulointerstitial fibrosis in obstructed kidneys via inhibition of EMT of tubular cells.

Recent reports indicate that a cluster of key microRNAs are highly expressed in kidneys [12] and that there are differences in the micro RNA expression profile in renal cortex versus medulla [13]; however, it was not until recently that specific roles of micro RNAs in renal function were investigated. These studies revealed that key micro RNAs can play roles in TGF-beta1 actions [14] and diabetic kidney disease [15] and that podocyte-specific deletion of Dicer, a key enzyme involved in micro RNA biogenesis, led to progressive glomerular and tubular damage along with proteinuria and other podocyte defects in mice [16].

Kato et al. demonstrated that the expression miR-192 is increased in renal glomeruli obtained from mouse models of type 1 and type 2 diabetes relative to corresponding control mice [14]. They also showed that the expression of miR-192 was increased by TGF-beta in mouse mesangial cells (MCs), whereas, conversely, the expression of its target, Zeb2, was decreased. This also paralleled in increased Collagen 1-alpha 2 (Col 1a2) and TGF-beta expression. These results suggested that the increase in TGF-beta in vivo in diabetic glomeruli and in vitro in MCs could induce miR-192 expression, which could target and down-regulate Zeb2 thereby to increase Col 1a2.

Several articles have shown that the miR-200 family targets ZEB1 and ZEB2 [1]–[4]. We also observed that Zeb1 and Zeb2 was a target of miR-200 family members that tended to be up-regulated by TGF-beta in kidney tubular cell lines. Thus, TGF-beta-induced increase in the expression of microRNAs (miR-192 and miR-200 family members) might coordinately down-regulate E-box repressors Zeb1 and Zeb2 to inhibit EMT and fibrosis of kidneys. Conversely, the down-regulation of ZEB1 and ZEB2 by TGF-beta via miR-200 family and miR-192 can affect upstream E-box regions [14].

Conversely, there are several reports that miR-200 family members and miR-192 can be down-regulated by TGF-beta, and this promotes EMT in cancer and other cell lines via subsequent up-regulation of targets ZEB1 and ZEB2 to repress E-cadherin [1]–[4]. Thus the effects of renal microRNAs may be cell type specific, and the microRNA signaling networks that mediate the effects of TGF-beta on MCs and epithelial cells and on metastatic and fibrotic EMT may not be identical.

Tubulointerstitial fibrosis is a common pathological event in the kidney during progressive renal injury and previous reports have shown that inhibition of tubulointerstitial fibrosis has a beneficial effect on kidney function [17]–[19]. We suggest that members of the miR-200 family, and miR-200b specifically, might constitute novel therapeutic targets in various kidney diseases.

Previous studies have shown that, in addition to TGF-beta, renal tubulointerstitial fibrosis can be induced by toll like receptor [18] and connective tissue growth factor [20] and that this effect can be opposed angiotensin II receptor blockers [21], statins [22], PPAR gamma agonists [23] and vitamin D [24]. We suggest that the therapeutic role of miR-200 family members be investigated in other models of kidney disease, particularly in the context of these factors. Finally, an alternative therapeutic approach in kidney disease may be to identify compounds that increase the expression levels of members of the miR-200 family of microRNAs.

### Conclusion

This study indicates that miR-200 family is up-regulated after ureter obstruction, miR-200b being strongly induced, and that miR-200b ameliorates tubulointerstitial fibrosis in obstructed kidneys.
